# The efficacy and safety of corticotomy and periodontally accelerated osteogenic orthodontic interventions in tooth movement: an updated meta-analysis

**DOI:** 10.1186/s13005-024-00409-1

**Published:** 2024-02-17

**Authors:** Huan Zhou, Yi-Fan Zhang, Yan-Xin Qi, Qian-Qian Zhang, Na Liu, Yue Chen

**Affiliations:** 1 Key Laboratory of Shaanxi Province for Craniofacial Precision Medicine Research, College of Stomatology, Xi’an Jiaotong University, Xi’an, China; 2 Department of Periodontology, College of Stomatology, Xi’an Jiaotong University, Xi’an, China; 3 Department of Prosthodontics, College of Stomatology, Xi’an Jiaotong University, Xi’an, China; 4 Department of Orthodontics, College of Stomatology, Xi’an Jiaotong University, Xi’an, China

**Keywords:** Orthodontics, Periodontal tissue, Tooth movement, Periodontal accelerated osteogenic orthodontics, Corticotomy

## Abstract

**Background:**

The surgically facilitated orthodontic strategy has been a promising strategy for orthodontic treatment recently. Therefore, the present meta-analysis was conducted to assess the available scientific evidence regarding the clinical outcomes, including the potential detrimental effects associated with these surgical procedures, with the aim of providing much more evidence-based information for clinical practice.

**Methods:**

An electronic search of three databases (PubMed, Cochrane, and Embase) and a manual search of relevant articles published up to May 2023 were carried out. Clinical trials (≥ 10 subjects) that utilized surgically facilitated orthodontic strategies with clinical and/or radiographic outcomes were included. Meta-analyses and sub-group analyses were performed to analyze the standardized mean difference (SMD) or weighted mean difference (WMD), and confidence interval (CI) for the recorded variables.

**Results:**

Nineteen studies published from Oct 2012 to May 2023 met the inclusion criteria. Based on the analysis outcomes, corticotomy treatment significantly decreased the alignment duration (WMD: -1.08 months; 95% CI = -1.65, -0.51 months, *P* = 0.0002), and accelerated the canine movement (WMD: 0.72 mm; 95% CI = 0.63, 0.81 mm, *P* < 0.00001) compared to the traditional orthodontic group. The periodontally accelerated osteogenic orthodontic (PAOO) strategy markedly reduced the total treatment duration (SMD: -1.98; 95% CI = -2.59, -1.37, *P* < 0.00001) and increased the bone thickness (SMD:1.07; 95% CI = 0.74, 1.41, *P* < 0.00001) compared to traditional orthodontic treatment.

**Conclusion:**

The present study suggests that facilitated orthodontic treatment in terms of corticotomy and PAOO strategy may represent attractive and effective therapeutic strategy for orthodontic patients.

**Supplementary Information:**

The online version contains supplementary material available at 10.1186/s13005-024-00409-1.

## Introduction

Traditional orthodontic treatment for malocclusions generally takes more than two years, because several factors can influence orthodontic treatment length, such as malocclusion type and severity, differences in interindividual biological response, clinician expertise, and patient compliance [1, 2]. Additionally, recent studies have demonstrated that bone anatomy, especially cortical bone thickness, negatively affects the overall duration of orthodontic treatment [2, 3]. While, prolonged orthodontic treatment duration may lead to various potential side effects on the teeth and surrounding tissues, such as dental caries, spot lesions, gingivitis, periodontitis and external apical root resorption, especially for orthodontic adult who demand aesthetic purposes, some of them may refuse orthodontic strategies due to the extended treatment duration [4–7].

Under this context, one primary concern for both clinicians and patients was to shorten the orthodontic treatment duration, and various methods of surgically facilitated orthodontic therapy, including corticotomy, piezocision, and the periodontally accelerated osteogenic orthodontic (PAOO) strategy, have been applied, and the clinical effectiveness of these procedures has been evaluated [8]. Corticotomy was proposed based on the “mechanical movement theory”; specifically, selective alveolar decortication (reduction in cortical bone density) leads to accelerated orthodontic tooth movement [9]. The “regional accelerated phenomenon” concept is the biological basis of the phenomenon of rapid tooth movement after bone injury [8, 10]. Currently, it is widely accepted that corticotomy can significantly decrease the orthodontic treatment duration, and the length, number, and depth of cortical bone incisions have been demonstrated to influence the acceleration rate of tooth movement [2]. Considering the invasive characteristics of corticotomy techniques, researchers have proposed flapless alternatives to traditional corticotomy procedure by utilizing piezoelectric devices, piezopuncture, or other micro-osteoperforations [11–14]. For example, piezocision represents a localized piezoelectric alveolar decortication technique that combines buccal microincisions and minimally invasive corticotomy that are performed with a piezotome [11, 15]. PAOO is a modification of corticotomy that combines periodontal and orthodontic treatment. Specifically, PAOO involves full-thickness flaps (buccal and/or lingual), osteotomies (in cortical bone), and bone grafts to ensure adequate periodontal support and to prevent the risks of bone dehiscence and/or fenestration [11, 16].

A variety of clinical studies have reported that these various facilitated orthodontic interventions can lead to promising results, such as decreasing orthodontic treatment time, facilitating impacted teeth eruption, opening bite correction, enhancing the resolution of crowding and increasing post-orthodontic stability [17–23]. At present, there is no consensus that these strategies can offer benefits to periodontal conditions, although some certain studies have reported a significant improvement in periodontal conditions in patients who received PAOO treatment [20]. However, these facilitated orthodontic strategies, such as PAOO, have been largely discredited for their invasive procedure and potential postoperative complications.

In this context, a comprehensive meta-analysis and systematic review assessing these various accelerated orthodontic techniques is necessary, although some reviews have been carried out that mainly focus on the clinical and/or radiographic outcomes of these facilitated orthodontic strategies. In addition, the majority of the current evidence-based records did not report any side effects, although certain data indicated the potential of a certain degree of periodontal injury [24, 25]. Therefore, a meta-analysis was carried out to thoroughly and critically compare the clinical outcomes, including the potential detrimental effects for patients who underwent facilitated orthodontic treatment to patients who were subjected to conventional orthodontic strategy, with the objective of providing much more evidence-based information for clinical practice.

## Methods

### Protocol and registration

Our meta-analysis was performed based on the Preferred Reporting Items for Systematic Reviews and Meta-analyses (PRISMA) statement [26] and the Guidelines of Cochrane Handbook for Systematic Reviews of Interventions Version 6.4 [27]. The present study was registered in the International Prospective Register of Systematic Reviews (PROSPERO, https://www.crd.york.ac.uk/prospero/, CRD42023410447).

### Search strategy

Three databases, including PubMed, EMBASE and Cochrane, were searched for published articles that assessed and compared the effects of various facilitated orthodontic treatment methods to conventional orthodontic treatment. The search terms included the following (“corticotomy” or “piezocision” or “periodontally accelerated orthodontic” or “PAOO” or “micro-osteoperforation” or “piezoelectric” or “corticision” or “accelerated” or “grafting” or “augmented” or “osteogenesis” or “osteogenic”) and (“orthodontics” or “orthodontic”). Additionally, the reference lists of the included studies and the related reviews were manually searched to explore any potentially relevant studies. The present study followed the PICOS structure, namely, population (P): subjects receiving orthodontic procedure to correct the malocclusion; intervention (I): corticotomy or PAOO strategy during orthodontic treatment; comparison (C): patients who were subjected to conventional orthodontic strategy; outcome (O): the clinical and/or radiographic outcomes; and study design (S): randomized or controlled clinical studies [28].

### Eligibility criteria

The inclusion criteria were as follows: (1) human randomized controlled trials (RCTs) or controlled clinical trials (CCTs); (2) studies comparing the outcomes of facilitated orthodontic treatment with conventional orthodontic treatment for patients (≥ 10 subjects); (3) studies reporting clinical outcomes clinical parameters (treatment duration, canine movement, keratinized gingival width, probing depth, plaque index, gingival index) and/or radiographic outcomes (bone thickness, bone density and root length) after the treatment; and (4) studies published in English and full-text available.

The exclusion criteria were as follows: (1) studies on pre-clinical models or in vitro; (2) studies with < 10 subjects; (3) non-comparative studies; (4) studies with insufficient information or data that could not be fully extracted; and (5) review articles, case reports, abstract editorials, commentaries, letters to the editor, monographs, and other study types.

### Literature search and study selection

A systematic literature search of all potential studies was carried out to identify all the relevant studies that were published up to May 2023. Specifically, two reviewers (YFZ, YXQ) searched the three databases and other relevant sources independently based on the search terms and then excluded duplicates. Then, three investigators (YXQ, QQZ, NL) screened and evaluated the titles and abstracts according to the eligibility criteria. The remaining studies that appeared to meet the aforementioned inclusion criteria were then subjected to full-text screening. Any disagreements on inclusion were resolved through discussion with all reviewers until a consensus was achieved.

### Data extraction

Two authors (YFZ, QQZ) extracted the relevant data from the included studies independently using a piloted and predefined data extraction tables, and then, all the authors re-checked and confirmed the obtained raw data. The following data were extracted from each study: (1) study characteristics, i.e., authors, publication year; (2) study design, i.e., sample size, research type, age range, malocclusion type; (3) intervention information, i.e., type of facilitated orthodontic treatment procedure, site of intervention, follow-up period; (4) outcomes i.e., clinical parameters (treatment duration, canine movement, keratinized gingival width, probing depth, plaque index and gingival index) and radiographic outcomes (bone thickness, bone density and root length) and, (5) methods of outcome measurements.

### Quality assessment

Risk of bias assessment for RCTs was carried out according to the guidelines of the Version 2 of Cochrane risk-of-bias tool for randomized trials (RoB 2) [29]. For CCTs, the Risk of Bias in Non-randomized Studies-of Interventions (ROBINS-I) tool was utilized to evaluate the risk of bias [30]. Two reviewers (HZ, YFZ) independently assessed the bias. For each domain and for the overall risk-of-bias judgement, the risk of bias was categorized as high (if one or more fields were assessed as “high risk of bias”), some concerns (for RCTs) or unclear (if at least one domain was assessed as “some concerns” or “unclear risk of bias”) or low (if all fields were assessed as “low risk of bias”). Any disagreement regarding the risk of bias was resolved through discussion with a third reviewer (YC).

### Data analysis

For the present meta-analysis, the combined effect size was expressed as the standardized mean difference (SMD) or the weight mean difference (WMD) with a 95% confidence interval (CI). *P* < 0.05 (two-tailed) was recognized to be statistically significant. The heterogeneity across studies was assessed with the *I*^2^ test, which ranged from 0 to 100%, and lower values represented less heterogeneity, while higher values indicated more heterogeneity. Additionally, sensitivity and sub-analyses were performed to identify the heterogeneity source as well as other potential confounding factors. RevMan 5.4.1 software (Cochrane Collaboration; www.cochrane.org/) was used for data analyses.

## Results

### Literature search & study characteristics

**Fig. 1:**
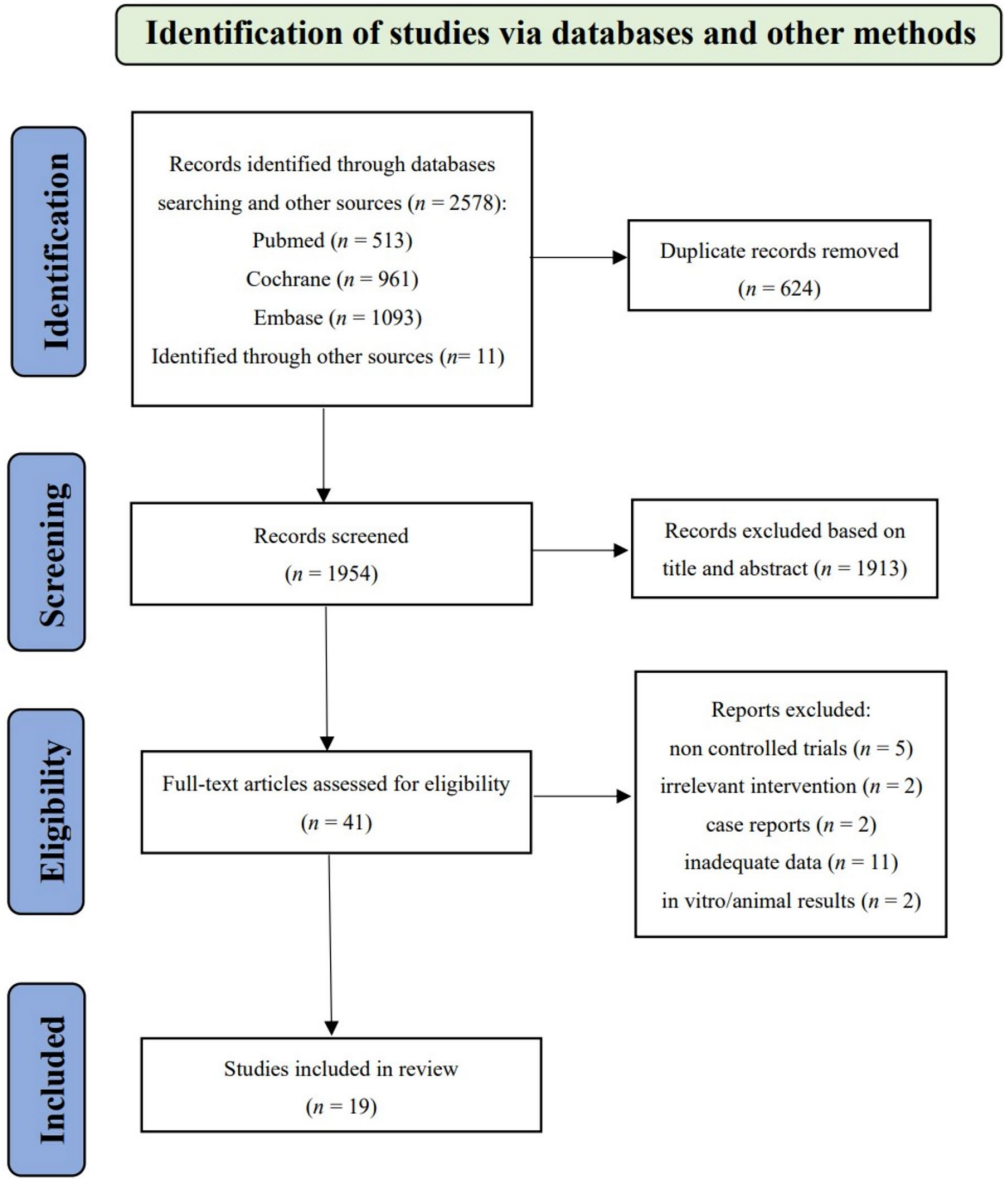
Flowchart of the enrolled studies

As illustrated in Fig. 1 and 2578 articles were initially identified according to the search strategy. After screening the titles and abstracts, 41 articles remained for further full-text evaluation. Then, 22 articles were further excluded based on the inclusion and exclusion criteria, in specific, no controlled trials (*n* = 5), irrelevant intervention (*n* = 2), case reports (*n* = 2), inadequate data (*n* = 11), and in vitro/animal results (*n* = 2). Ultimately, this meta-analysis included 19 full-text articles published up to Apr, 2023.

 Of the 19 articles that enrolled 634 patients, eight compared corticotomy to conventional orthodontic treatment, eight articles compared PAOO to conventional orthodontic treatment, and three articles compared corticotomy to PAOO treatment. Among the enrolled studies, 11 were RCTs, and 8 were CCTs. The age of the participants ranged from 14 to 42 years. The main characteristics of the included studies were summarized in Table 1.

**Table 1:** Characteristics of the included studies

Author /year	Trail design	Age (Mean ± SD /Range) (years)	Case type/Treatment Location	Follow up (months)	Groups/Sample size	Duration (months)	Bone Thickness Changes (mm)	Root Length Changes (mm)	KGW (mm)	Periodontal Parameter (PD/GI/PI)	Canine crown movement (mm)
Ma (2023) [31]	CCT	21.05 ± 2.66	Class III/lower anterior teeth	15.65 vs. 23.3 m post-treatment	T:PAOO *n* = 18 C:Conventional Orthodontics *n* = 18	T:15.65 ± 4.13 C:23.3 ± 4.52	T:0.58 ± 0.71 C:-0.14 ± 0.3	T:-1.16 ± 1.06 C:-0.82 ± 0.64	N/A	N/A	N/A
Wang (2022) [32]	CCT	23.43 ± 3.63	Class II/anterior teeth	24.43 vs. 31.16 m post-treatment	T:PAOO *n* = 13 C:Conventional Orthodontics *n* = 13	T:24.43 ± 2.53 C:31.16 ± 4.17	N/A	N/A	N/A	N/A	N/A
Wu (2015) [33]	CCT	20.35 ± 1.79	Class III/upper anterior teeth	12.48 vs. 18.87 m post-treatment	T:PAOO *n* = 12 C:Conventional Orthodontics *n* = 12	T:12.48 ± 2.17 C:18.87 ± 4.17	N/A	N/A	N/A	N/A	N/A
Sirri (2020) [34]	RCT	21.4 ± 1.63	Mild or moderate crowding /lower anterior teeth	3.83 vs. 5.24 m post-treatment	T:Corticotomy *n* = 30 C:Conventional Orthodontics *n* = 30	T:3.82 ± 0.52 C:5.24 ± 0.45	N/A	N/A	N/A	PD T:0.63 ± 0.37 C:0.65 ± 0.36 GI T:0.24 ± 0.22 C:0.16 ± 0.2 PI T:0.78 ± 0.24 C:0.61 ± 0.12	N/A
Sultana (2022) [35]	RCT	21.0 ± 2.58	Severe crowding /upper anterior teeth	2 m post-treatment	T:Corticotomy *n* = 6 C:Conventional Orthodontics *n* = 7	T:4.05 ± 0.6 C:5.09 ± 0.73	N/A	N/A	N/A	PD T:0.08 ± 0.12 C:0.07 ± 0.06	N/A
Uribe (2017) [36]	RCT	29.7 ± 11.0	moderate crowding /lower anterior teeth	7 m post-treatment	T:Corticotomy *n* = 16 C:Conventional Orthodontics *n* = 13	T:3.35 ± 1.14 C:3.67 ± 1.51	N/A	N/A	N/A	N/A	N/A
Ahn (2016) [37]	CCT	22.29 ± 4.94	Class III/lower anterior teeth	8.7 vs. 10.9 m post-presurgical orthodontic	T:PAOO *n* = 15 C:Conventional Orthodontics *n* = 15	N/A	T:-0.12 ± 0.48 C:-0.44 ± 0.31	T:-0.6 ± 0.59 C:-0.67 ± 0.63	N/A	N/A	N/A
Jing (2020) [38]	CCT	22.6	Class III/anterior teeth	6 m after surgery	T:PAOO *n* = 47 C:Conventional Orthodontics *n* = 13	N/A	T:0.55 ± 0.64 C:-0.25 ± 0.23	N/A	T:0.34 ± 1.20 C:0.17 ± 1.22	N/A	N/A
Xu (2020) [39]	CCT	18–30	Class III/upper anterior teeth	6 m after surgery	T:PAOO *n* = 10 C:Conventional Orthodontics *n* = 10	N/A	T:0.04 ± 0.46 C:-0.29 ± 0.23	N/A	T:0.35 ± 0.77 C:0.25 ± 0.87	N/A	N/A
Kilinc (2023) [40]	RCT	17.30 ± 2.23	moderate crowding /lower anterior teeth	4 m post-treatment	T:Corticotomy *n* = 15 C:Conventional Orthodontics *n* = 15	N/A	N/A	N/A	N/A	PD T:0.03 ± 0.31 C:0.03 ± 0.27 GI T:0.08 ± 0.53 C:0.42 ± 0.5 PI T:0.3 ± 0.64 C:0.44 ± 0.55	N/A
Wilcko (2015) [41]	CCT	29.9 ± 12.2	Class I/II/III/lower anterior teeth	19.4 vs. 15.9 m post-treatment	T:PAOO *n* = 35 C:Conventional Orthodontics *n* = 35	T:7.1 ± 1.7 C: 22.1 ± 6.8	N/A	N/A	N/A	N/A	N/A
Wang (2013) [19]	CCT	24.2 ± 3.6	Class III /lower anterior teeth	7.8 vs. 13.3 m post-treatment	T:PAOO *n* = 26 C:Conventional Orthodontics *n* = 30	T:7.8 ± 4.2 C:13.3 ± 3.5	N/A	N/A	N/A	N/A	N/A
Aksakalli (2016) [42]	RCT	16.3 ± 2.4	Class II/canine	3.54 vs. 5.59 m post-treatment	T:Corticotomy *n* = 10 C:Conventional Orthodontics *n* = 10	N/A	N/A	N/A	N/A	N/A	T:2.90 ± 0.86 C: 1.73 ± 0.72
Alfawal (2018) [4]	RCT	18.08 ± 3.5	Class II/upper anterior teeth	3 m after surgery	T:Corticotomy *n* = 17 C:Conventional Orthodontics *n* = 17	N/A	N/A	N/A	N/A	N/A	T:3.57 ± 0.48 C:2.7 ± 0.27
Çağlı (2021) [43]	RCT	14–22	Class II/upper anterior teeth	3 m after surgery	T:Corticotomy *n* = 12 C:Conventional Orthodontics *n* = 12	N/A	N/A	N/A	N/A	PD T:-0.02 ± 0.01 C:-0.02 ± 0.01 PI *P* > 0.05 GI *P* > 0.05	T:2.88 ± 0.13 C:2.19 ± 0.11
Raj (2020) [44]	RCT	23.18 ± 1.41	Class II/canine	6 m after surgery	T:Corticotomy *n* = 20 C:Conventional Orthodontics *n* = 20	N/A	N/A	N/A	N/A	PD T:-0.05 ± 0.15 C:-0.06 ± 0.58	N/A
Shoreibah (2012) [45]	RCT	24.5	Moderate crowding /lower anterior teeth	6 m after surgery	T1:Corticotomy *n* = 10 T2:PAOO *n* = 10	N/A	Bone density(%) T1:-17.60 ± 5.77 T2:25.85 ± 15.64	T1:-0.06 ± 0.03 T2:-0.05 ± 0.03	N/A	PD T1:-1.43 ± 0.24 T2:-1.56 ± 0.61	N/A
Bahammam (2016) [46]	RCT	21.2 ± 1.43	Moderate crowding/lower anterior teeth	9 m after surgery	T1:Corticotomy *n* = 11 T2:PAOO *n* = 11	N/A	Bone density(%) T1:0.87 ± 15.28 T2:22.85 ± 16.87	T1:-0.03 ± 0.67 T2:-0.04 ± 0.53	N/A	PD T1:-0.36 ± 0.09 T2:-0.37 ± 0.08	N/A
Agrawal (2018) [47]	RCT	21.9 ± 2.13	Class I & II/canine	t1 Before orthodontic treatment t2 post- treatment	T1:Corticotomy *n* = 10 T2:PAOO *n* = 10	N/A	N/A	T1:-0.2 ± 0.26 T2:-0.14 ± 0.36	N/A	N/A	N/A

*m* Month, *T* Treatment group, *C* Control group, *RCT* Randomized controlled trial, *CCT* Controlled clinical trials, *PAOO* Periodontally accelerated osteogenic orthodontics, *KGW* Keratinized gingival width, *PD* Pocket depth, *PI* Periodontal index, *GI* Gingival index

### Risk of bias

**Fig. 2:**
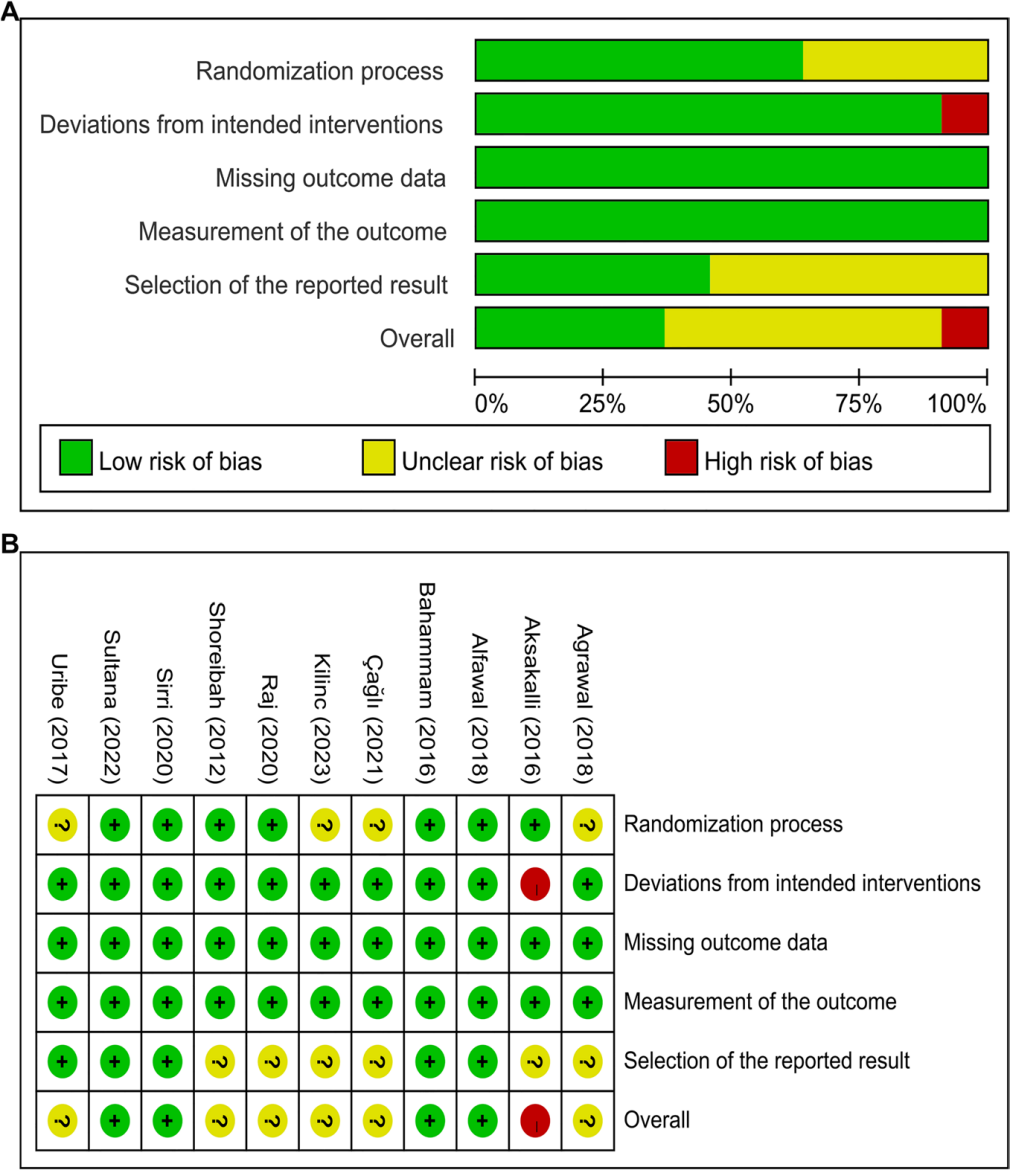
Risk of bias assessment. **A** Risk of bias item presented as percentages across all included randomized clinical trials (RCTs). **B** Graph of the risk of bias for the RCTs

**Fig. 3:**
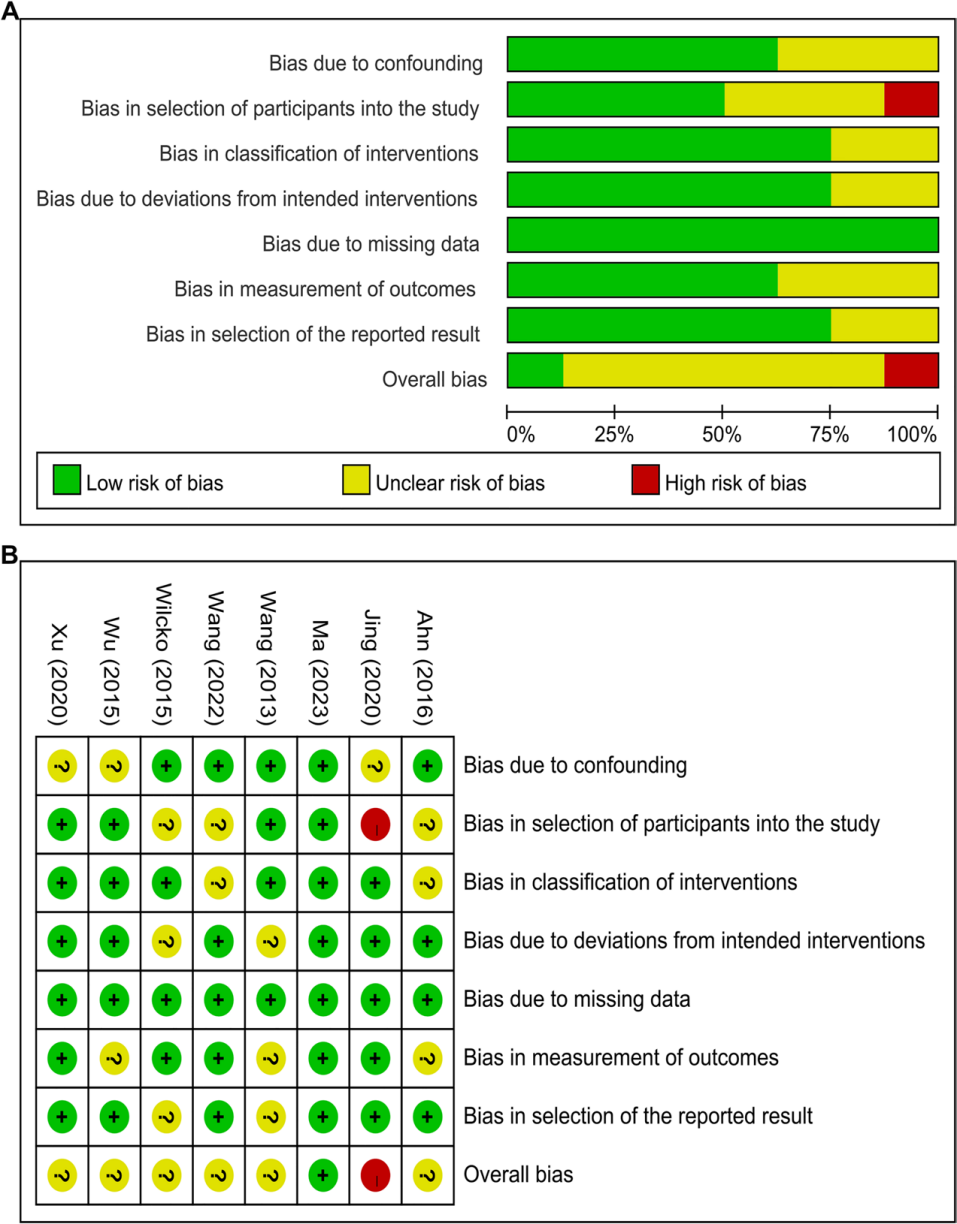
Risk of bias assessment. **A** Risk of bias item presented as percentages across all included controlled clinical trials (CCTs), **B** Graph of the risk of bias for the CCTs.

Quality assessments for all enrolled articles were performed. The details of the risk of bias are illustrated in Figs. 2 and 3. Specifically, for RCTs (Fig. 2), one study was ranked as “high risk of bias” for “Deviations from intended interventions”, four studies were categorized as “some concerns” for “Randomization process”, and six studies were categorized as “some concerns” for “Selection of the reported result of bias”.

For CCTs (Fig. 3), one study was categorized as “high risk of bias” for “Bias in selection of participants into the study”. In addition, seven studies were categorized as “unclear risk of bias” for lacking information on other bias assessment domains except the “Bias due to missing data”. Thus, the included CCTs were judged to possess a considerable risk of bias.

### Outcome measurements

#### Meta-analyses for the outcomes of corticotomy compared to traditional orthodontic treatment

##### Alignment duration

**Fig. 4:**
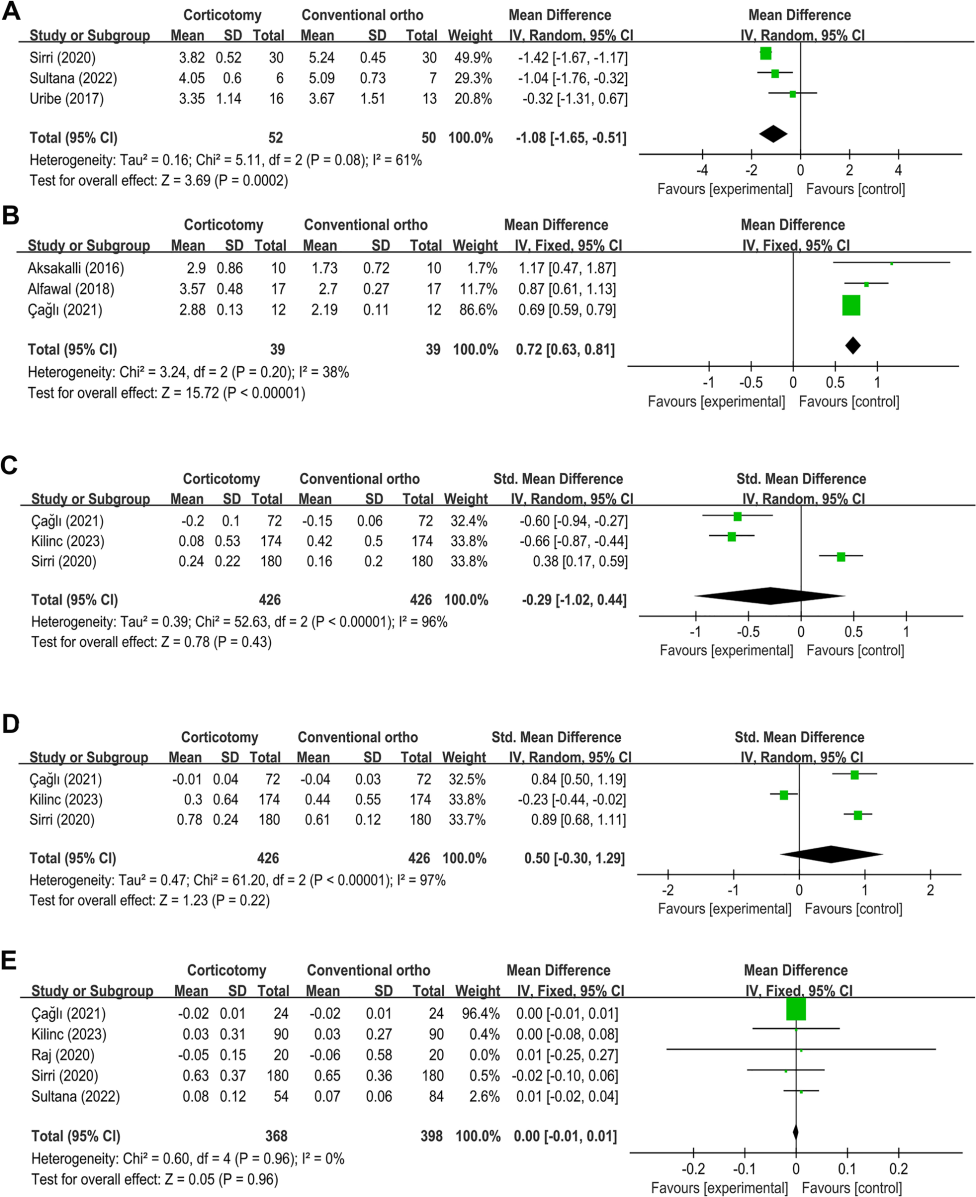
Forest plot shows the mean effect size and 95% confidence interval (CI) for the changes of alignment duration (**A**), canine movement (**B**), gingival index (**C**), plaque index (**D**), and probing depth (**E**) outcomes of corticotomy compared to conventional orthodontic treatment

Three studies reported data on the alignment duration [34, 35], and the WMD was − 1.08 months (95% CI = -1.65, -0.51 months, *P* = 0.0002), favoring the corticotomy treatment. While, the comparison among included studies demonstrated a moderate heterogeneity (I^2^ = 61%) (Fig. 4A).

### Canine movement

Three studies reported data on the canine movement [4, 42, 43], and the meta-analysis exhibited a WMD of 0.72 mm (95% CI = 0.63, 0.81 mm, *P* < 0.00001), favoring the corticotomy strategy. The comparison among the enrolled studies demonstrated low heterogeneity (I^2^ = 38%) (Fig. 4B).

### Gingival index

Three studies reported data on the gingival index [34, 40, 43], and according to the meta-analysis, there was no significant difference between the groups, for the gain in SMD was − 0.29 (95% CI= -1.02, 0.44, *P* = 0.43), and the heterogeneity among the included studies was high (I^2^ = 96%) (Fig. 4C).

### Plaque index

Additionally, three studies reported data on the plaque index [34, 40, 43], the SMD of the pooled studies was 0.50 (95% CI= -0.30, 1.29, *P* = 0.22), representing no statistically significant difference between the groups, and there was a high heterogeneity among the three studies (I^2^ = 97%) (Fig. 4D).

### Probing depth

Five studies reported data on the probing depth [34, 35, 40, 43, 44], and according to the meta-analysis, there was no significant difference between the surgically facilitated orthodontic treatment and conventional, because the gain of WMD was 0.00 mm (95% CI= -0.01, 0.01 mm, *P* = 0.96), and there was a low heterogeneity among the enrolled studies (I^2^ = 0%) (Fig. 4E).

### Meta-analyses for the outcomes of PAOO compared to traditional orthodontic treatment

#### Total treatment duration

**Fig. 5:**
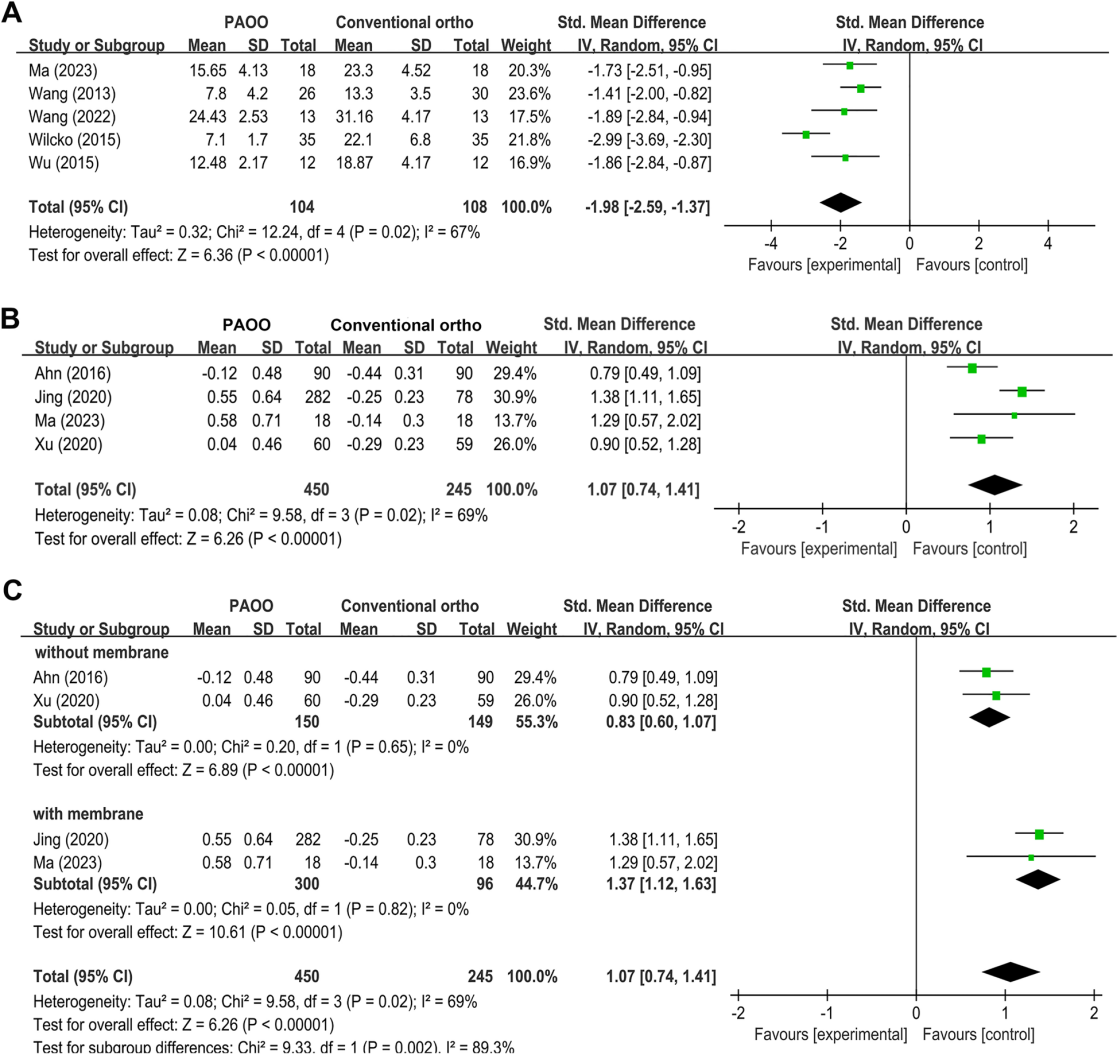
Forest plot shows the mean effect size and 95% confidence interval (CI) for the changes of total treatment duration (**A**) and bone thickness (**B**, **C**) outcomes of periodontally accelerated osteogenic orthodontic compared to conventional orthodontic treatment

Five studies reported data on the total treatment duration [19, 31–33, 41], and the SMD was − 1.98 (95% CI = -2.59, -1.37, *P* < 0.00001). While, the comparison among the included studies demonstrated a high heterogeneity (I^2^ = 67%) (Fig. 5A).

### Bone thickness

For the four studies reporting data on the gain of bone thickness [31, 37–39], the SMD of the pooled studies was 1.07 (95% CI = 0.74, 1.41, *P* < 0.00001), favoring the PAOO strategy; however, there was significant heterogeneity (I^2^ = 69%) (Fig. 5B). In addition, sub-group analysis for bone thickness outcomes based on bone graft methods (with or without membrane) was performed, and according to the results (Fig. 5C), the gain in SMD of bone thickness between studies with or without membrane was significant (*P* = 0.002), favoring the bone graft with membrane.

#### Meta-analyses for the outcomes of corticotomy compared to the PAOO strategy

##### Root length

**Fig. 6:**
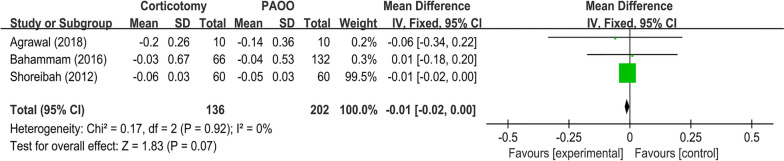
Forest plot shows the mean effect size and 95% confidence interval (CI) for the changes of root length outcome of corticotomy compared to periodontally accelerated osteogenic orthodontic treatment

For the three studies that reported data on the root length [45–47], there was no significant difference between the corticotomy and PAOO groups, as the WMD was − 0.01 mm (95% CI = -0.02, 0.00 mm, *P* = 0.07), and there was low heterogeneity among the included studies in terms of root length (I^2^ = 0%) (Fig. 6).

#### Qualitative analysis

For those enrolled studies (including keratinized gingival width, root length, probing depth, bone density) less than three, qualitative analysis was performed.

#### Keratinized gingival width

 Two studies compared the change in keratinized gingival width between PAOO and traditional orthodontic treatment [38, 39], As shown in Table 2, although more keratinized gingival width gain was identified in PAOO group (0.34 mm vs. 0.17 mm; 0.35 mm vs. 0.25 mm, respectively) in relative to traditional orthodontic treatment, the difference was not significant.

**Table 2:** Qualitative analysis results of keratinized gingival width

Study ID	Groups	Surgical intervention	Outcomes	Results	keratinized gingival width	Conclusion
Jing (2020) [38]	T: PAOO *n* = 47 C: Conventional Orthodontics *n* = 13	T1: Full-thickness flaps were elevated on the labial aspect, and vertical alveolar decortication was performed with bone grafting. T2: traditional orthodontic treatment	keratinized gingival width	More keratinized gingival gain was found in the T group, but the difference was not significant (0.34 vs. 0.17 mm).		PAOO guided tissue regeneration and increased keratinized gingival width, but the change between two groups was not significant.
Xu (2020) [39]	T: PAOO *n* = 10 C: Conventional Orthodontics *n* = 10	T1Full-thickness flaps were elevated, and vertical interproximal cortical bone incisions were created with bone grafting. T2: traditional orthodontic treatment	keratinized gingival width	More keratinized gingival gain was found in the T group, but the difference was not significant (0.35 vs. 0.25 mm).		Although the postoperative width was greater at 6 months, the changes in both groups were not statistically significant.

*PAOO* Periodontally accelerated osteogenic orthodontic

(red, C > T; green,T > C; yellow, no significant difference)

#### Root length

 Two studies reported and compared the data on the root length between PAOO and traditional orthodontic treatment [31, 37]. According to the qualitative analysis outcome, there was no significant difference between the PAOO and conventional groups (-0.6 mm vs. -0.67 mm; -1.16 mm vs. -0.82 mm, respectively) (Table 3).

**Table 3:** Qualitative analysis results of root length

Study ID	Groups	Surgical intervention	Outcomes	Results	Root length	Conclusion
Ahn (2016) [37]	T: PAOO *n* = 15 C: Conventional Orthodontics *n* = 15	T1: Full-thickness flaps were elevated, vertical interproximal cortical bone incisions were created with bone grafting. T2: traditional orthodontic treatment	root length	Root length decreased in T Group by 0.6 mm vs. 0.67 mm in C Group. There were no significant differences in their changes between the groups.		PAOO in presurgical orthodontic treatment does not increase the risk of root resorption.
Ma (2023) [31]	T: PAOO *n* = 18 C: Conventional Orthodontics *n* = 18	T1: Full-thickness flaps were elevated, and with a vertical releasing incision added at the premolar area, circumscribing the corticotomy with bone grafting. T2: traditional orthodontic treatment	root length	Root length decreased in T Group by 1.16 mm vs. 0.82 mm in C Group. There were no significant differences in their changes between the groups.		There was no statistically significant difference between the groups in regards to root resorption.

*PAOO* Periodontally accelerated osteogenic orthodontic

(red, C > T; green,T > C; yellow, no significant difference)

### Bone density and probing depth

 As illustrated in Table 4, Two studies indicated that the PAOO can increase the bone density (25.85% vs. -17.60%; 22.85% vs. 0.87%, respectively) when compared with the corticotomy strategy [45, 46]. In addition, two studies compared data on probing depth between PAOO and corticotomy treatment [45, 46], and according to the qualitative analysis, there was no significant difference (-1.56 mm vs. -1.43 mm; -0.37 mm vs. -0.36 mm, respectively) between the two groups (Table 4).

**Table 4:** Qualitative analysis results of bone density and probing depth

**Study ID**	**Groups**	**Surgical intervention**	**Outcomes**	**Results**	**Bone density**	**Probing depth**	**Conclusion**
Shoreibah (2012) [45]	T1:Corticotomy *n* = 10 T2:PAOO *n* = 10	T1: Full-thickness flaps were reflected, and vertical decortication was performed. T2: Full-thickness flaps were reflected,and vertical decortication was performed combined with bone grafting.	Probing depth Bone density	Bone density decreased in T1 by 17.60% vs. an increase of 25.85% in T2. Probing depth decreased in T1 Group by 1.43 mm vs. 1.56 mm in T2 Group.			PAOO increased the alveolar bone density, and there was no significant difference between the groups in regards to probing depth.
Bahammam (2012) [46]	T1:Corticotomy *n* = 11 T2: PAOO *n* = 22	T1: Full-thickness flaps were reflected,and vertical decortication was performed. T2: Full-thickness flaps were reflected, and vertical decortication was performed combined with bone grafting.	Probing depth Bone density	Bone density increase of 0.87% in T1 vs. an increase of 22.85% in T2. The probing depth of T1 decreased 0.36 mm vs. 0.37 mm in T2.			PAOO increased the alveolar bone density, there was no significant differences in probing depth changes between the two groups.

*PAOO* Periodontally accelerated osteogenic orthodontic

(red, T1 > T2; green, T2 > T1; yellow, no significant difference)

### Sensitivity analysis

Sensitivity analysis was also carried out to assess the robustness and stability of the meta-analysis results (reported by more than three studies). Fig. S1 exhibited that the circles corresponding to the included studies were located near the middle vertical line where the combined effect size was located. It appeared that no studies had a significant impact on the combined effect size. Besides, after systematically removing one study at a time, and recalculating the pooled results, we found that there was no significant change after sensitivity analysis for probing depth (corticotomy compared to traditional orthodontic treatment), total treatment duration (PAOO compared to traditional orthodontic treatment), or bone thickness (PAOO compared to traditional orthodontic treatment), respectively. Therefore, for the pooled MD, neither probing depth, total treatment duration nor bone thickness outcome were significantly affected by any study.

## Publication bias

Statistical analysis of publication bias was not performed, because fewer than 10 studies were included in all the quantitative syntheses.

## Discussion

This comprehensive meta-analysis including 19 articles with 634 patients was carried out, with the aim of evaluating the efficacy and safety of corticotomy and PAOO treatment techniques. Based on the analysis outcomes, the corticotomy strategy can significantly decrease the alignment duration and accelerate canine movement. In addition, PAOO procedure markedly reduced the total treatment duration and increased the bone thickness.

It is well known that conventional orthodontic treatment may lead to a variety of side effects in terms of decalcification, dental caries, gingival inflammation or recession, pain and discomfort, and apical root resorption, because it usually takes approximately two years to complete when treating moderate to severe malocclusion [48, 49]. Hence, various methods of accelerating orthodontic strategy, including corticotomy and PAOO treatment, have been introduced and utilized, which were demonstrated to accelerate tooth movement and reduce the treatment duration [50]. Consistent with previous studies, our present meta-analysis also demonstrated that these two surgically facilitated orthodontic strategies can shorten the total duration and/or the alignment duration, and accelerate canine movement, which was mainly attributed to the regional acceleratory phenomenon that allows for demineralization at surgical sites as well as the adjacent bone, and then an enhanced bone response that permits localized tissue remodeling, finally leading to accelerated healing that is 2–10 times that of physiological healing [46, 51].

Additionally, a gain in bone thickness was observed in our meta-analysis, which mainly resulted from the grafting material within the PAOO treatment. This procedure is usually utilized in cases with a thin buccal bone, where bone grafting materials in terms of bone derivative material and bioabsorbable collagen membranes deliver a benefit to the surrounding soft and hard tissues, to transfer the bone from thin type to a more robust type [51, 52]. In addition, it was verified that bone grafting materials can reduce the risk of bone fenestration, bone dehiscence and gingival recession within the orthodontic procedure [20, 51]. Our previous study found that the PAOO strategy was beneficial to periodontal conditions in orthodontic patients with bone dehiscence and fenestration, where the proportion of teeth with a thick gingival phenotype increased from 33.61% at baseline to 53.13% at the end of the follow-up; additionally, the bone thickness was significantly increased compared to the baseline [20].

Root resorption is a common phenomenon within orthodontic treatment and is related to many factors. A previous study reported that the corticotomy procedure can result in a 44% average increase in root resorption compared with the control group [25]. However, Charavet C and colleagues demonstrated that the increase in root resorption did not exist in either corticotomy or the conventional group [11, 53]. Our meta-analysis demonstrated that there was no significant difference in root length between the PAOO and corticotomy treatment, and the qualitative analysis indicated that the difference between the PAOO and conventional group was not significant. Therefore, based on our present results, accelerating orthodontic treatment did not increase the risk of root resorption, which was consistent with the previous analysis [51]. In addition, our meta-analysis demonstrated that the changes in periodontal parameters in terms of probing depth, plaque index, and gingival index in the group subjected to facilitated orthodontic treatment were not significantly different relative to the conventional group, and these results may have resulted from the strict oral hygiene measures applied to the patients. Most of the studies that evaluated periodontal parameters post-accelerating orthodontic procedures did not find adverse effects on periodontal tissues. These results, together with our meta-analysis, indicated that surgically accelerated interventions were safe for periodontal tissue [11, 53].

For those enrolled studies less than three, qualitative analysis was performed in our study. Two studies pooled in the present study demonstrated that there was no significant difference in the gain of keratinized gingival width, although there was a tendency towards keratinized tissue gain after the PAOO procedure compared with conventional orthodontic treatment. For the two studies, one reported that augmented corticotomy-facilitated orthodontic (PAOO) treatment resulted in a significant gain in keratinized gingival width [38]. Xu X reported that although the keratinized gingival width was greater at six months after surgery, the difference between the two groups was not statistically significant [39]. Besides, qualitative analysis suggested that PAOO treatment can increase the bone density when compared with the corticotomy strategy, while, the change of data on probing depth between the two groups was not significant treatment [45, 46]. Considering the small sample size and the relative short duration of the follow-up, future standardized RCTs with large samples are required to explore the effect of augmented corticotomy strategy on the keratinized gingival width, bone density and probing depth.

According to previous studies, scars were observed in 50% of the patients receiving corticotomy treatment, although patient satisfaction was markedly higher in the corticotomy group than that in the control group; therefore, caution should be taken when corticotomy is implemented in patients with a high smile line because the risk of slight scarring exists [11, 22, 53, 54]. In addition, several studies assessed tooth vitality, and there have been no reported cases of loss of tooth vitality until now [35, 55, 56], while, comparisons between these studies were not achievable because different evaluation methods were applied. Moreover, it should be noted that orthodontic treatment may fail if the dentist concentrates on occlusion/function only, while overlooking the acceptance and perceptions of the patient, because the orthodontic therapy requires patient compliance. It was reported that fear from the surgery (53.2%) and fear from pain (36.9%) were the top two reasons for not selecting corticotomy-assisted orthodontics [57], thus, the level of patient’s acceptance to these surgically facilitated orthodontic strategy should be valued, considering the possible swelling and pain postoperatively as well as other concerns.

The present meta-analysis has several limitations. First, the heterogeneity within the included studies (design and methodology) was moderate to high, and the risk of bias of the included studies was considerable due to the bias of confounding factors within certain CCTs, and the lack of information for the randomization process and selection of the reported result within RCTs. Second, the follow-up of certain enrolled studies was 3–6 months post-operation, and a relatively longer follow-up period of the response of periodontal soft and hard tissues to these facilitated orthodontic strategies was lacking. Third, other confounding factors in terms of bone grafts, types of assessment methods, and language restrictions probably have an effect on the analysis outcomes, Thus, the results of the meta-analysis should be interpreted with caution. Before these facilitated orthodontic procedures can be fully utilized in daily clinical practice, reliable conclusions should be obtained from further well-designed RCTs. Therefore, there is an urgent need for high-quality clinical studies conducted with additional attention given to the study design, outcome measurement methodology and especially the safety as well as the potential adverse effects.

## Conclusion

Within the limitations of the present meta-analysis, facilitated orthodontic treatment in terms of corticotomy and PAOO strategy may represent effective therapeutic approach for orthodontic patients because the corticotomy strategy can significantly decrease the alignment duration and accelerate canine movement. The PAOO procedure can markedly reduce the total treatment duration and increase the bone thickness. In addition, according to our meta-analysis and the available body of literature, facilitated orthodontic treatment including corticotomy and PAOO strategy was safe for periodontal tissues, as no major post-operative side reactions were reported, and there was not sufficient scientific evidence to support the absence or presence of clinically relevant post-treatment adverse effects. While, the results of the present meta-analysis should be interpreted with caution because of the short-term follow-up, the inadequate sample of participants, and the heterogeneity of the studies.

### Supplementary Information

**Additional file 1.**

## Data Availability

All data generated or analyzed during this study are included in the article.

## Abbreviations

RCTs: Randomized clinical trials
CCTs: Controlled clinical trials
CI: Confidence interval
SMD: Standardized mean difference
WMD: Weight mean difference
PRISMA: Preferred reporting items for systematic reviews and meta-analyses
PAOO: Periodontally accelerated osteogenic orthodontic
