# Supplementary material for: The efficacy and safety of corticotomy and periodontally accelerated osteogenic orthodontic interventions in tooth movement: an updated meta-analysis
Source: Head Face Med. 2024 Feb 17;20:12. doi: 10.1186/s13005-024-00409-1 (PMC10874089; doi:10.1186/s13005-024-00409-1)
Supplement: Supplementary file 1 — Additional file 1. [file 13005_2024_409_MOESM1_ESM.docx]

Fig. S1 Sensitive analysis of included studies (more than three studies) for probing depth (A), total treatment duration (B), and bone thickness (C).


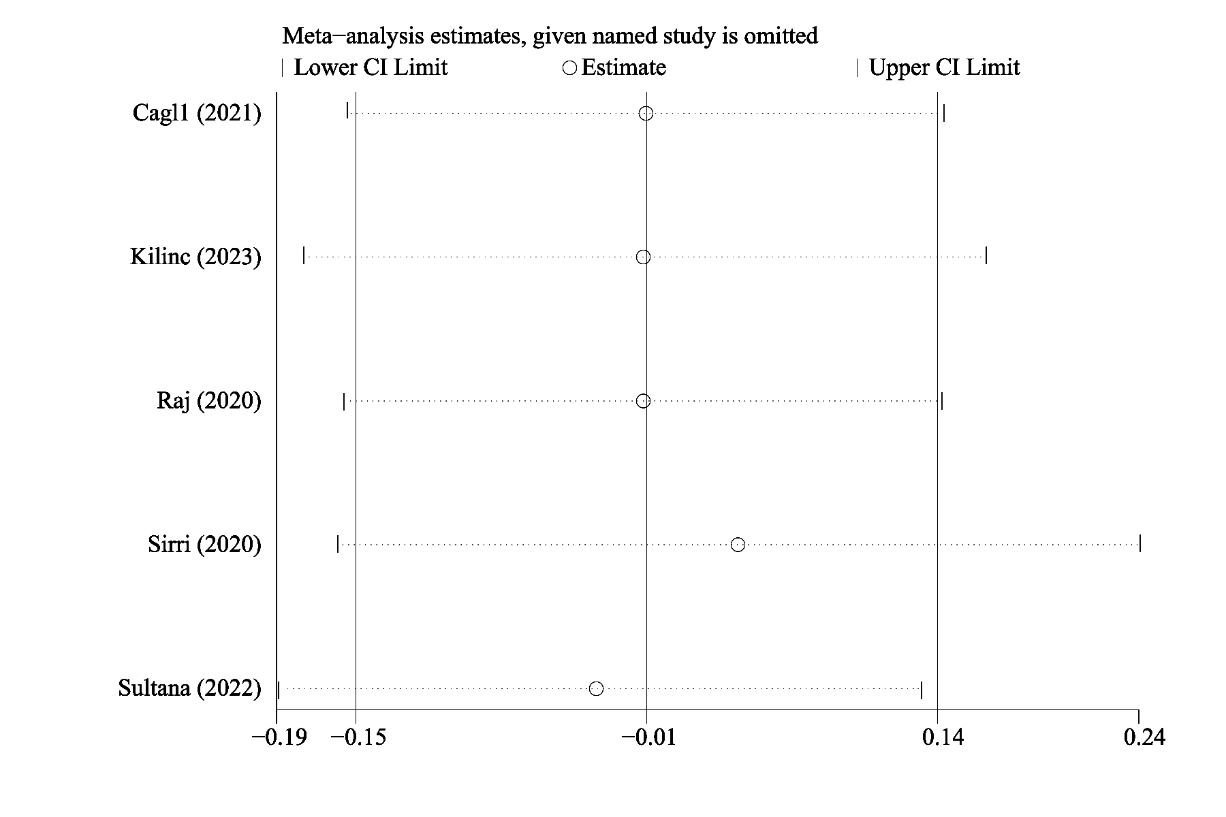


**A**: Sensitive analysis of included studies for probing depth (corticotomy compared to traditional orthodontic treatment)


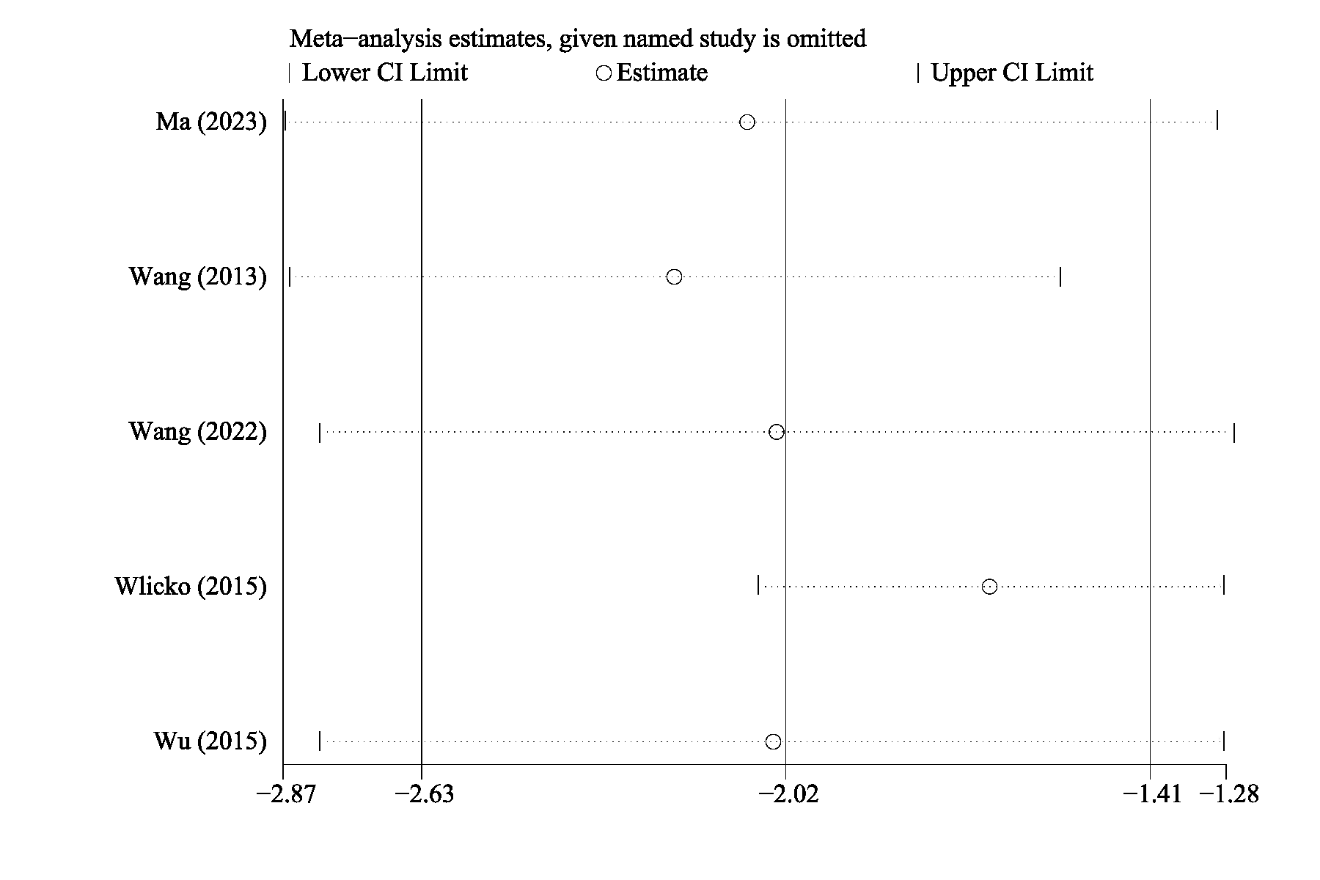


**B**: Sensitive analysis of included studies for total treatment duration (periodontally accelerated osteogenic orthodontic compared to traditional orthodontic treatment)


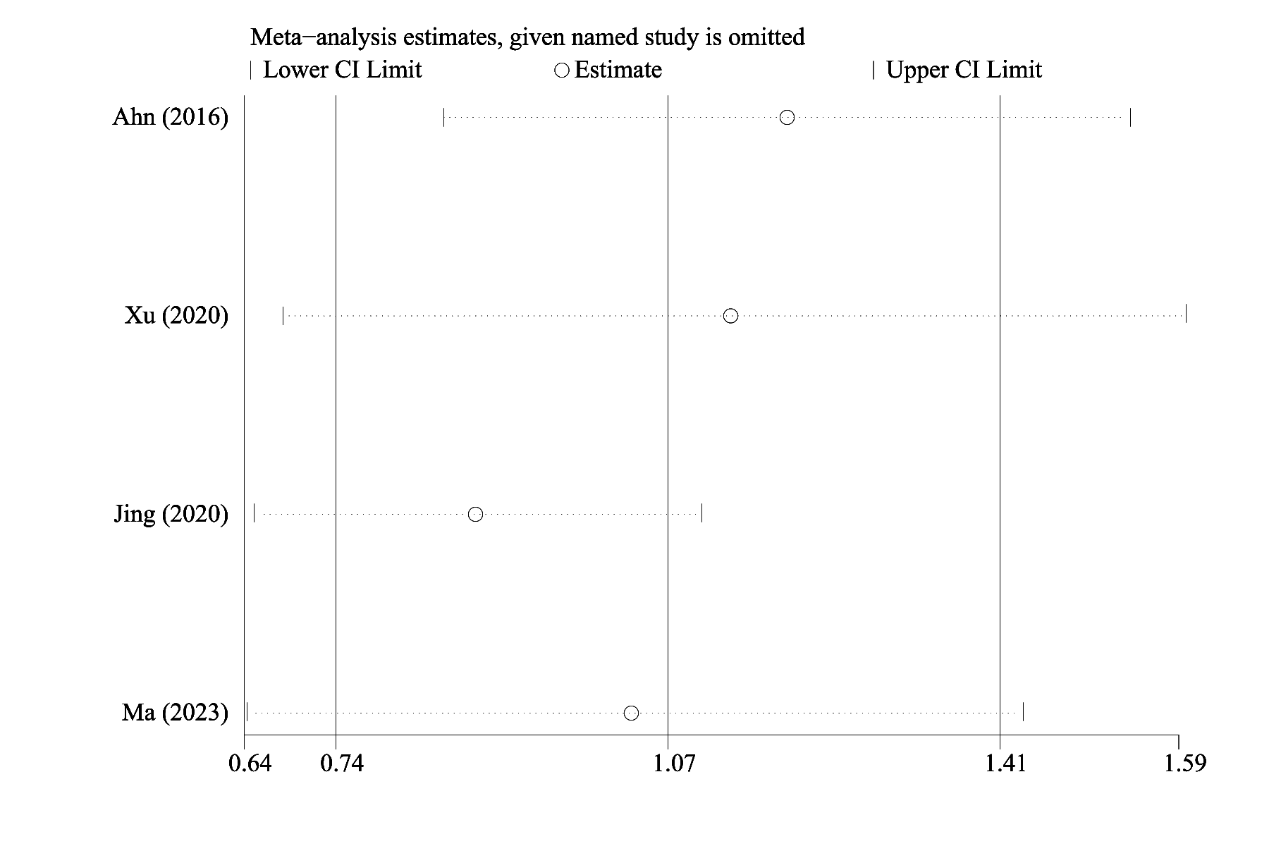


**C**: Sensitive analysis of included studies for bone thickness (periodontally accelerated osteogenic orthodontic compared to traditional orthodontic treatment)
